# Fostering Professionalism and Addressing Antisemitism at Medical School Commencements

**DOI:** 10.5041/RMMJ.10545

**Published:** 2025-04-29

**Authors:** Anas Babar

**Affiliations:** Final Year MBBS, King Edward Medical University, Lahore, Pakistan

**Keywords:** Antisemitism, medical education, professionalism

**Dear Editor**,

I read with great interest the article titled “US Medical Schools’ 2024 Commencements and Antisemitism: Addressing Unprofessional Behavior” by Roth and Wald.^1^ The study highlights a concerning trend of antisemitic regalia, offensive signage, and disruptive protest behaviors at the graduation ceremonies of top-ranked US medical schools. Such incidents raise ethical and professional concerns, undermining the solemnity of commencement events and compromising the core principles of the medical profession, including respect, inclusivity, and impartiality.

Commencement ceremonies symbolize the culmination of years of rigorous medical training and the transition to a profession that prioritizes empathy and compassion for all individuals, regardless of background. The display of antisemitic themes or disruptive protests during these events not only tarnishes the image of medical education institutions but also risks eroding public trust in healthcare professionals.^2^ Furthermore, as noted by Papadakis et al., there is a strong correlation between unprofessional behavior during medical training and future disciplinary actions by medical licensing boards, underscoring the long-term implications of such conduct.^3^

The recommendation by Roth and Wald to enforce stricter regalia policies and uphold professionalism at commencements is crucial. However, such measures should be complemented by proactive educational interventions. Incorporating sensitivity training, historical education on antisemitism, and modules on respectful discourse within medical curricula can foster a culture of empathy and inclusivity.^4^ The adoption of the “4 Es” framework—Education, Engagement, Empathy, and Enforcement—provides a structured approach to combating antisemitism and similar forms of discrimination in medical education.^1^

To ensure long-lasting change, it is essential to create safe spaces within medical institutions for open discussions on sensitive issues while upholding standards of professionalism. Additionally, faculty must serve as role models by exemplifying respectful communication and ethical conduct. The integration of these strategies can help medical schools produce healthcare professionals who embody the values of integrity, respect, and cultural sensitivity, which are essential for delivering high-quality patient care.

As the healthcare landscape becomes increasingly diverse, it is imperative for academic medical institutions to take decisive steps in addressing all forms of discrimination, including antisemitism, and promoting professionalism. By doing so, they can uphold the sacred trust bestowed upon the medical profession and ensure a more inclusive and respectful environment for both patients and practitioners.
